# Role of staging laparoscopy for gastric cancer patients

**DOI:** 10.1002/ags3.12283

**Published:** 2019-08-21

**Authors:** Takeo Fukagawa

**Affiliations:** ^1^ Department of Surgery School of Medicine Teikyo University Tokyo Japan

**Keywords:** chemotherapy, gastric cancer, laparoscopy, peritoneal dissemination, staging

## Abstract

Staging laparoscopy (SL) is frequently carried out in patients with advanced gastric cancer. However, some clinical questions are being debated and consensus must be obtained. With this aim, a literature search of PubMed/MEDLINE was carried out using the keywords “gastric cancer,” “SL,” and “diagnostic laparoscopy”. Articles published online up to February 2019 were analyzed, focusing on the following questions. (i) What is an adequate indication for SL? (ii) How do you carry out SL? (iii) Does SL provide accurate information about peritoneal dissemination? (iv) Is the yield of SL different by tumor location? (v) Is SL a safe procedure? (vi) Is “repeat SL” needed? (vii) Does SL provide oncological benefit? Results provided the following responses: (i) In Western countries, clinically resectable advanced tumor is an indication for SL. Terms to be introduced for adequate indication include “location,” “type 4 (linitis feature),” “large tumor,” “equivocal computed tomography (CT] findings,” and “lymph node swelling”. (ii) Exploration of the entire peritoneal cavity is preferable. (iii) Detection rate of peritoneal disease is 43%‐52% in Japanese institutions and 7.8%‐40% in other countries. False‐negative findings during SL were 0%‐17%, and 10%‐13% when limited to cytology. (iv) Yield of SL was higher in gastric cancer compared with esophagogastric junctional tumor. (v) SL‐related complications were estimated to occur in 0.4%. (vi) Repeat SL is important after treatment. (vii) If the efficacy of neoadjuvant chemotherapy for patients with P0CY1 is established, SL can provide oncological benefit. SL can be carried out safely and effectively. Considering the prevalence of neoadjuvant treatment, the role of SL will become more important.

## 1 INTRODUCTION

Staging laparoscopy (SL) has been incorporated into the diagnostic strategy for advanced gastric cancer (GC) for years. In some therapeutic guidelines,^1^, ^2^, ^3^, ^4^ SL is recommended for preoperative staging. Historically, the value of SL has been controversial. In 1985, Shandall and Johnson wrote, “In gastric carcinoma, the value of laparoscopy is doubtful as a high percentage requires at least palliative surgery”.^5^ In contrast, Gross et  al wrote, “Laparoscopy is a useful method for the assessment of GC and allows easy biopsy, particularly of peritoneal deposits. Unnecessary laparotomy is avoided, and the morbidity of the procedure is minimal”.^6^

**Figure 1:**
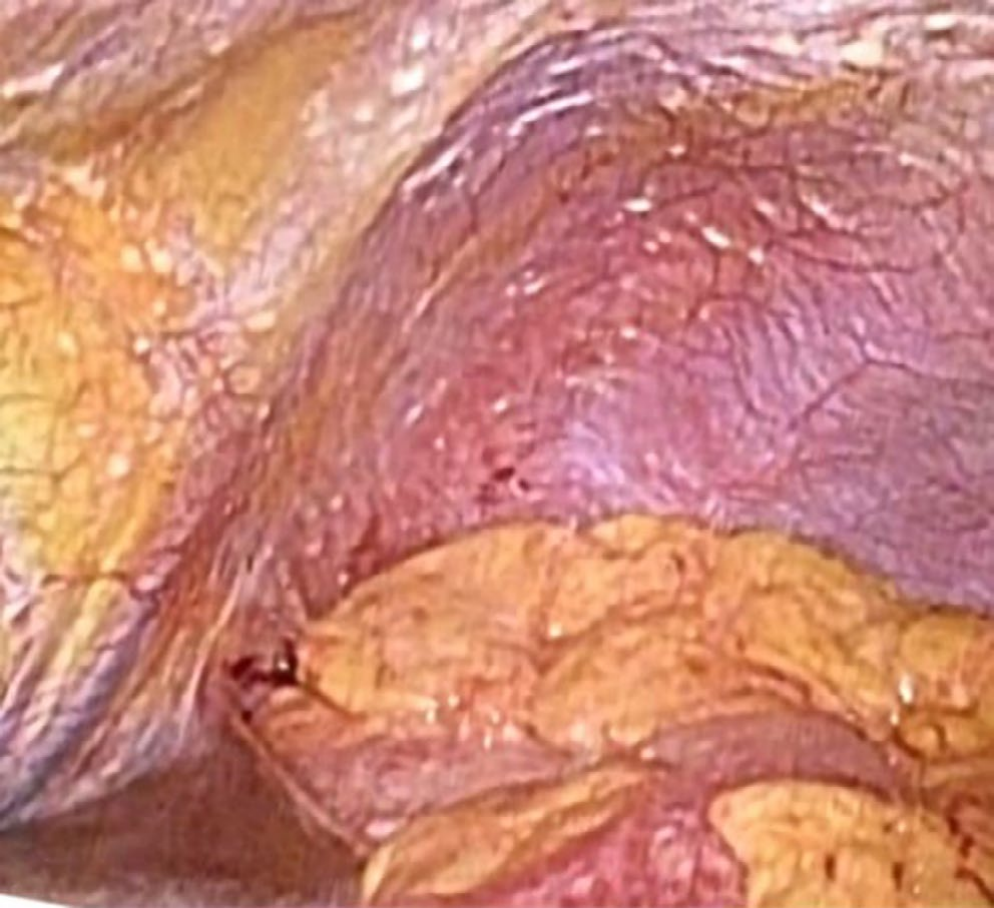
Peritoneal dissemination during staging laparoscopy. Small nodules of peritoneal dissemination on the surface of diaphragm

The main purpose of SL is to detect occult peritoneal dissemination (Figure 1), aiming for more accurate M1 staging (distant metastases) than diagnosis by imaging. Peritoneal dissemination (P) may be diagnosed using a computed tomography (CT) scan with findings of ascites and multiple mesenteric or omental nodules, but diagnostic accuracy is not high.^7^ Peritoneal dissemination is sometimes detected only during laparotomy, without definitive radiological findings. The presence of peritoneal dissemination, regarded as stage IV GC, is a poor prognostic factor. Patients have no indication for gastrectomy except for bleeding or obstruction, and undergo systemic chemotherapy.^8^ If those patients undergo chemotherapy without palliative gastrectomy, laparotomy would be non‐therapeutic. SL can provide accurate information about peritoneal dissemination and lavage cytology (CY) with less surgical invasiveness and an appropriate therapeutic strategy.^9^ It is beneficial to avoid useless laparotomies and shorten the time between diagnosis and the initiation of chemotherapy. However, some problems regarding this procedure should be discussed.

## 2 METHODS

The present review was based on articles from PubMed and MEDLINE and carried out in February 2019. “Gastric cancer”, “staging laparoscopy” and “diagnostic laparoscopy” were used as search terms. After a full‐text search for 79 articles published after 2000, a final set of 41 studies was extracted with a sample size of patients who underwent SL larger than 50. If not satisfied with this condition, the articles including important information were included in this review.

This review was constructed to answer the following clinical questions: (i) What is an adequate indication for SL? (ii) How do you carry out SL? (iii) Does SL provide accurate information about peritoneal dissemination? (iv) Is the yield of SL different by tumor location of either esophagogastric junctional cancer (EGJ) or gastric cancer (GC)? (v) Is SL a safe procedure? (vi) Is “repeat SL” needed? (vii) Does SL provide oncological benefit?

## 3 RESULTS

Results provided responses to the seven questions based on the database search.

### 3.1 What is an adequate indication for SL?

The main purpose of SL is to detect occult peritoneal disease (P and/or CY) that cannot be definitively diagnosed using imaging examinations.^10^ In Western countries where advanced gastric cancer is common, a reported indication for SL was “resectable GC and EGJ without definite distant metastases”. In many Japanese institutions, SL is carried out based on the clinical trials of the Japan Clinical Oncology Group (JCOG). JCOG0501^11^ is a randomized controlled trial evaluating the efficacy of neoadjuvant chemotherapy (NAC) for patients with large type 3 (≥8 cm) and type 4 advanced gastric cancer. JCOG 0405^12^ is a phase II clinical trial evaluating the efficacy of NAC for GC patients with bulky lymph node metastases (≥3 cm) or para‐aortic node metastases (PAN). In both clinical trials, SL was mandatory for the confirmation of eligibility criteria. Therefore, “large type 3 and type 4” or “bulky N/PAN” are frequently adopted in practice as indications for SL.

Indications for SL are discussed in two ways (Table 1). First, some clinical factors that lead to a high incidence of peritoneal disease among SL cases are evaluated using multivariate analysis. Sarela et al^13^ reported the incidence of peritoneal dissemination by clinical findings: EGJ (42%), whole stomach (66%), poorly differentiated adenocarcinoma (36%), age ≤70 (34%), lymphadenopathy ≥1 cm by CT scan (49%), and depth of tumor was T3 or T4 (63%). Among these factors, “location (EGJ or whole stomach)” and “lymphadenopathy by CT scan” were significant predictive factors by multivariate analysis of 65 SL cases. Ikoma et al^14^ reported similar results for location: fundus/body/antrum (38%), poorly differentiated (38%), signet ring cell morphology (41%), linitis feature (66%), and equivocal CT findings (65%). Among them, “poorly differentiated”, “linitis feature” and “equivocal CT findings” were significant by multivariate analysis. The second way of discussing indications for SL is a validation method using a large number of cases, including patients who did not undergo SL. Tsuchida et al^15^ determined that “three portions (=whole stomach),” “type 3/4/5” and “lymph node metastases by CT scan” were significant predictive factors for peritoneal disease by multivariate analysis of 31 SL cases. If the indication for SL was defined as two or three factors among these, sensitivity, specificity, positive predictive factor (PPV), negative predictive factor (NPV) and accuracy for peritoneal disease were 91.9%, 37.9%, 46.7%, 88.7% and 58.0%, respectively, using a total of 231 cases limited to c T3/T4. The study of Hu et al^16^ used a similar method to Tsuchida.^15^ The significant predictive factors for peritoneal disease were “≥4 cm”, “T4b” and “type 3 or 4”. If the indication for SL was defined as two or three factors among them, sensitivity, specificity, PPV, NPV and accuracy for peritoneal disease were 85%, 69%, 43%, 94% and 72%, respectively, using a total of 582 cases (c T2‐4b). The report by Hur et al,^17^ however, was not an analysis of SL cases. “Type 3 or 4”, “T3 or T4” and “≥4 cm” were significant by multivariate analysis using 589 clinically advanced GC cases. If the indication for SL was defined as “all three factors”, 42.4% of all cases were expected to be indicated for SL, and sensitivity, specificity, PPV, NPV and accuracy for peritoneal dissemination were 83.3%, 63.2%, 24.0%, 96.4% and 65.7%, respectively, using the same series. In the report of Irino et al,^18^ the indication for SL was defined as “large type 3 (≥8 cm)” or “type 4” or “bulky N” or “PAN” or “suspicious findings of peritoneal disease”. Validation analysis using 721 cases (c T3/4) in the same period showed that sensitivity, specificity, PPV, NPV and accuracy for peritoneal disease were 67.6%, 76.5%, 47.5%, 88.2% and 74.3%, respectively. When the validation was analyzed with 2213 cases (c T1‐4), the results changed to 66.1%, 93.4%, 47.5%, 96.9% and 91.5%, respectively.

**Table 1:** Indications for staging laparoscopy (SL) for gastric cancer patients

First author	Case	No. (Validation)	No. (SL)	P(M1) prevalence by clinical findings	Indication: multivariate analysis and validation
Sarela^13^	Resectable GC & EGJ	65	65	GEJ (42%), whole stomach (66%), poorly differentiated (36%), age ≤70 (34%), lymphadenopathy ≥1 cm (49%), T3/T4 (63%)	Location (GEJ or whole stomach)
					Lymphadenopathy by CT
Ikoma^14^	Resectable GC & EGC	662	662	Fundus/body/antrum (38%), poorly differentiated (38%), signet ring cell morphology (41%), linitis feature (66%), equivocal CT findings (65%)	Poorly differentiated
					Linitis feature
					Equivocal CT findings
Tsuchida^15^	T4 (SE,SI)	231	31	Tumor location: 3 portions (64%),	Location (3 portions)
				≥8cm (49%)	Macroscopic type (3/4/5)
				Macroscopic type: 3/4/5 (43%), T4b (59%)	Lymph node metastases by CT
				Lymph node metastases (40%)	
					“2 or 3 factors” (169/231, 73.2%)
					Sensitivity 91.9%, specificity 37.9%,
					PPV 46.7%, NPV 88.7%, accuracy 58.0%
Hu^16^	T2‐4b	582	582	≥4 cm (43%)	≥4 cm
				Middle third involved (31%)	T4b
				T4b (52%)	Type 3/4
				Type 3 (34%), type 4 (41%)	
					“2 or 3 factors” (249/582, 42.8%)
					Sensitivity 85%, specificity 69%
					PPV 43%, NPV 94%, accuracy 72%
Hur^17^	Clinically advanced GC	589	0	T3 (15%) T4 (39%), N1 (11%) N2 (20%)	Type 3 or 4
				≥4 cm–<8 cm (11%), ≥8 cm (26%), type 3 (13%) type 4 (35%), undifferentiated (14%), anterior wall involved (17%), posterior wall involved (16%)	T3 or T4
					≥4 cm
					“all three factors” (250/589, 42.4%)
					Sensitivity 83.3%, specificity 63.2%,
					PPV 24%, NPV 96.4%, accuracy 65.7%
Irino^18^	c T3/T4	721	156	Large type 3 (56%), type 4 (53%)	Large type 3 or type 4
				Bulky nodes/PAN swelling (21%)	Bulky nodes or PAN swelling
				Suspicion of peritoneal disease (20%)	Suspicion of P
					“Any of these factors” (246/721, 34%)
					Sensitivity 67.6%, specificity 76.5%,
					PPV 47.5%, NPV 88.2%, accuracy 74.3%
	c T1‐4	2213			“Any of these factors” (246/2213, 11%)
					Sensitivity 66.1%, specificity 93.4%,
					PPV 47.5%, NPV 96.9%, accuracy 91.5%

CT, computed tomography; EGJ, esophagogastric junction; GC, gastric cancer; GEJ, gastroesophageal junction; NPV, negative predictive value; PAN, para aortic lymph node; PPV, positive predictive value.

### 3.2 How do you carry out SL?

Surgical procedure for SL has been established, and it was similar in each study. It must be determined as to whether exploration inside the omental bursa is mandatory. If GC was located at the posterior side of the stomach, there could be peritoneal dissemination inside the omental bursa only. Several studies described inspection inside the omental bursa,^13^, ^15^, ^19^, ^20^, ^21^, ^22^, ^23^ but the incremental detection of peritoneal dissemination was not described. We also questioned whether the total length of mesentery must be inspected. Ishigami et al^20^ and Miki et al^24^ included “the surface of the entire bowel” and “from the oral to anal side “in the exploration area of SL, but there was no description in other reports. Definite answers could not be drawn from the reported articles, but wide exploration may be needed to reduce false‐negative results.

### 3.3 Does SL provide accurate information about peritoneal dissemination?

Reports on the detection of P1 and/or CY1 that were not seen by imaging examinations are listed in Table 2. Ikoma et al^14^ defined “the yield of SL” as the proportion of patients among all patients who underwent laparoscopy for staging whose laparoscopy showed positive findings, including those with macroscopic carcinomatosis, positive cytology, or other clinically important findings. The main purpose of SL was to find disseminated nodules that could not be detected by imaging, but other clinical findings that could change the therapeutic strategy (such as liver metastases or invasion to adjacent organs) may be detected at SL. Irino et al,^18^ Hosogi et al,^25^ Miki et al,^24^ Ishigami et al,^20^ Yamagata et al^19^ and Nakagawa et al^26^ reported the detection rate of P1 and/or CY1 as 47%, 45%, 53.4%, 42.7%, 46% and 51.6%, respectively. Results of these studies from Japanese institutions were almost similar (range: 42.7% to 53.4%), and higher than those from other countries (range: 7.8% to 40%).^16^, ^21^, ^23^, ^27^, ^28^, ^29^, ^30^, ^31^, ^32^, ^33^, ^34^ The reason for this discrepancy was the difference in the indication for SL. In Japanese institutions, the indication for SL was adjusted to meet the clinical trial eligibility of JCOG (large type 3 & type 4). This indication was targeted to this special group with a high possibility of peritoneal disease among the patients with advanced GC.

**Table 2:** Detection rate of P1 and/or CY1 during staging laparoscopy

First author	Country	Year of publication	Period	Cases (over 50)	Indication for SL	Yield
Irino^18^	Japan	2018	2003‐2013	156	Large type 3 & type 4, bulky N/PAN, suspicious for P	47%
Hosogi^25^	Japan	2017	2006‐2015	120	≧5 cm and/or bulky N	45%
Strandby^27^	Denmark	2016	2010‐2012	219	Resectable GC & EGJ (EGJ: 78%)	7.80%
Ikoma^14^	USA	2016	1995‐2012	711	Resectable GC & EGC (EGJ: 43.2%)	36%
Simon^28^	France	2016	2005‐2011	116	Resectable GC, EGC & EC, ≧T3 or N+	12.90%
Hu^16^	China	2016	2004‐2014	582	GC ≧T2	25.80%
Miki^24^	Japan	2015	2008‐2014	88	Large type 3 & type 4	53.40%
Convie^29^	UK	2015	2007‐2013	295	Resectable GC, EGC & EC	21.40%
Mirza^30^	UK	2015	1996‐2013	378	Resectable GC & EGJ, potential future NAC	13.70%
Tourani^31^	Australia	2015	1999‐2010	148	GC ≧T2	25.60%
Ishigami^20^	Japan	2014	–	178	GC ≧T2	42.70%
Bhatti^32^	Pakistan	2014	2005‐2012	149	Resectable GC & EGJ	40%
Munasinghe^21^	UK	2013	2006‐2010	316	Resectable GC & EGJ	22.50%
Yamagata^19^	Japan	2012	2001‐2009	124	Large type 3 & type 4, cN+, suspicious for P	46%
Kapiev^33^	Israel	2010	–	78	Resectable GC & EGJ	29.50%
Nakagawa^26^	Japan	2007	1999‐2005	93	Resectable GC T3‐4 (SE/SI)	51.60%
Sarela^13^	USA	2006	1993‐2002	657	Resectable GC & EGJ	23%
Burke^34^	USA	1997	1990‐1995	103	Resectable GC	31%
Lowy^23^	USA	1996	1991‐1995	71	Resectable GC	23.20%

EC, esophageal cancer; EGJ, esophagogastric junction; GC, gastric cancer; NAC, neoadjuvant chemotherapy; PAN, para‐aortic node metastases; SL, staging laparoscopy; –, not listed.

“False negative for SL” is listed in Table 3. In the case of P0/P0CY0 during SL, following surgery with curative intent was attempted after several weeks. In the case of P0CY1, surgery was carried out to address symptoms (bleeding, obstruction or institutional indication). P1 or CY1 was sometimes confirmed following laparotomy, which was classified as “false negative for SL”. Limited to macroscopic disseminated nodule, the rate of false negativity was reported as 0% to 17.2%. From a similar indication for SL (large type 3, type 4, and suspicious for dissemination), Irino et al,^18^ Miki et al^24^ and Yamagata et al^19^ reported the rate of false negativity as 11%, 17.2% and 15.6%, respectively. This indication implied a strong possibility of dissemination, so SL should be meticulously and widely carried out for various sites in the peritoneal cavity. In the reports from Western countries,^13^, ^21^, ^22^, ^23^, ^33^, ^34^, ^35^, ^36^, ^37^, ^38^ the rate of false negativity was lower than that from Japan.^15^, ^18^, ^19^, ^24^, ^25^ This is also due to differences in the indication for SL.

**Table 3:** Diagnostic accuracy during staging laparoscopy: False negative

First author	False negative	Indication
Irino^18^	11% (7/66)	Large type 3 & type 4, bulky N/PAN, suspicious for P
Hosogi^25^	5.9% (1/17)	≧5 cm and/or bulky N
Miki^24^	17.2% (5/29)	Large type 3 & type 4
Munasinghe^21^	0% (0/183)	Resectable GC & EGJ
Cardona^35^	1.9% (3/155)	Repeat SL
Yamagata^19^	P: 15.6% (10/64) CY: 13.3% (6/45)	Large type 3 & type 4, cN+, suspicious for P
Tsuchida^15^	6.7% (1/15)	c T4M0
Kapiev^33^	0% (0/55)	Resectable GC & EGJ
Shimizu^39^	CY: 10% (1/10)	Large type 3 & type 4, bulky N/PAN
Muntean^22^	6.5% (2/31)	Resectable GC
de Graaf^40^	8.1% (27/332) (resectable to unresectable)	Resectable GC & EGJ
Nakagawa^26^	44.4% (4/9) (p0cy1 to p1)	Resectable GC T3‐4 (SE/SI)
Sarela^13^	10% (41/401) (p: 56%)	Resectable GC & EGJ
Lavonius^36^	10.7% (3/28)	Resectable GC
Asencio^37^	4.5% (2/44)	Resectable GC
Burke^34^	8.4% (6/71, M1)	Resectable GC
Stell^38^	7.1% (4/56)	Resectable GC
Lowy^23^	7.3% (3/41, M1)	Resectable GC

EGJ, esophagogastric junction; GC, gastric cancer; PAN, para‐aortic node metastases; SL, staging laparoscopy.

CY0 at initial SL may change to CY1 at the following laparotomy. Yamagata et al^19^ and Shimizu et al^39^ reported a false‐negative result for CY as 13.3% and 10%, respectively. For cytological examination during SL, the stirring of lavage fluid could be insufficient for adequate quality and quantity of cell collection. Munasinghe et al^21^ reported that the routine use of subphrenic cytology in combination with pelvic lavage during SL had an incremental benefit in detecting positive CY compared to either pelvic or subphrenic cytology alone. Nakagawa et al^26^ noted that 44.4% (4/9) of patients with P0CY1 during initial SL were reassessed as P1 following laparotomy. Patients with P0CY1 diagnosed during SL may have hidden disseminated nodules which are also the target of chemotherapy.

Peritoneal disease can be confirmed by SL, but the extent of local invasion from GC could not be completely explored. Diagnosis may change from “resectable” to “unresectable” following laparotomy not only as a result of peritoneal dissemination but also as a result of unexplored local invasion during SL. de Graaf et al^40^ reported this conversion rate as 8.1%. Reason for the “unresectable” diagnosis was locally advanced disease (59.3%) and metastases (40.7%, peritoneal, liver, and others). When direct invasion of GC to the pancreas was suspected by CT scan, exploration inside the omental bursa is needed for more accurate information during SL.

### 3.4 Is the yield of SL different by tumor location of either esophagogastric junctional cancer or gastric cancer?

Some studies about SL from Western countries included not only GC, but also EGJ cancer or lower esophageal cancer (including squamous cell carcinoma) for the indication of SL. Yield of SL was higher in cases with GC compared with EGJ in all studies except one (Table 4).

**Table 4:** Yield of staging laparoscopy: Tumor location

First author	Country	Lower esophagus	EGJ		GC	Total
Strandby^27^	Denmark		5.3% (9/171)	<	16.7% (8/48)	7.80%
Simon^28^	France	0% (0/24)	12.2% (5/41)	<	19.6% (10/51)	12.90%
Convie^29^	UK		16.2% (22/136)	<	25.8% (41/159)	21.40%
Bhatti^32^	Pakistan		28%	<	48%	40%
Munasinghe^21^	UK	14.5% (20/138)	13.9% (5/36)	<	32.4% (46/142)	22.50%
Mirza^30^	UK		12.7% (27/212)	<	14.8% (26/175)	13.70%
Sarela^13^	USA		23.8% (25/105)	=	22.5% (124/552)	23%

EGJ, esophagogastric junction; GC, gastric cancer.

### 3.5 Is SL a safe procedure?

Staging laparoscopy is considered a safe procedure carried out within one hour under general anesthesia. Some reported intestinal injury during SL.^28^, ^29^, ^39^, ^41^ In many reports, there were no SL‐related complications (Table 5), but the estimated SL‐related complication rate was 0.4% based on total accumulated data.

**Table 5:** Complications related to staging laparoscopy

First author	SL‐related complications		All perioperative complications	
Irino^18^	0% (0/156)		0.6% (1/156)	Angina
Hu^16^	0% (0/62)		3.2% (2/62)	Pneumonia
Marmor^41^	2.8% (4/145)	Intestinal injury (2), liver laceration, air embolus	6.2% (9/145)	CD grade I/II/III/IV:
				4/3/1/1
Simon^28^	0.8% (1/116)	Intestinal injury	0.8% (1/116)	
Munasinghe^21^	0% (0/316)		0.3% (1/316)	AMI
Convie^29^	0.3% (1/317)	Intestinal injury	0.3% (1/317)	
Yamagata^19^	0% (0/124)		0% (0/124)	
Tsuchida^15^	0% (0/31)		0% (0/31)	
Kapiev^33^	0% (0/78)		0% (0/78)	
Shimizu^39^	2.9% (1/34)	Intestinal injury	2.9% (1/34)	
Muntean^22^	–		2.2% (1/45)	
de Graaf^40^	0% (0/416)		0% (0/416)	
Nakagawa^26^	0% (0/93)		0% (0/93)	
Burke^34^	–		4.2% (1/24)	
Total	0.4% (7/1888)		0.9% (18/1957)	

AMI, acute myocardial infarction; SL, staging laparoscopy; –, not listed.

### 3.6 Is “Repeat SL” after chemotherapy needed?

“Repeat SL” refers to a second SL carried out after chemotherapy. Thiels et al^42^ reported a 12% positive rate at “repeat SL” after chemotherapy for patients who were classified as P0/CY0 during the first SL done before chemotherapy. If the response of chemotherapy was poor, disseminated nodules could be growing during chemotherapy. “Repeat SL” had the benefit of preventing non‐therapeutic laparotomies. Cardona et al^35^ reported 5.5% positive and 1.9% false‐negative rates at “repeat SL”. Nakamura et al^43^ described conversion surgery based on the diagnosis of “second‐look SL”. They carried out conversion surgery if “second‐look SL” confirmed that P1/CY1 converted to P0/CY0 after induction chemotherapy. When conversion surgery is more prevalent, SL after treatment will become very important.

### 3.7 Does SL provide oncological benefit?

Detection of occult peritoneal disease can avoid non‐therapeutic laparotomy and shorten the interval to induction of chemotherapy. However, the survival benefit was obscured because the therapeutic strategy of systemic chemotherapy without gastric resection was the same in both groups. Burke et al^34^ reported that there was no significant difference in the survival of patients with unresectable M1 disease between undergoing laparoscopy only and undergoing laparotomy only.

Do patients who are cytology positive only without macroscopic peritoneal dissemination (P0CY1) during SL survive longer by induction chemotherapy than patients with POCY1 who immediately underwent surgery after diagnosis? In their review, Jamel et al^44^ reported that “patients with initial positive cytology may have a good prognosis following neoadjuvant treatment if the cytology results change to negative after treatment”. Badgwell et al^45^ also reported that some patients with P0CY1 achieved long‐term survival and could be considered for neoadjuvant treatment prior to attempts at surgical resection. This problem should be argued in terms of whether NAC provided survival benefit for patients with P0CY1.

## 4 DISCUSSION

A Canadian review team^46^ published a systematic review about the accuracy and indications for diagnostic laparoscopy prior to curative‐intent resection of GC. They reported accuracy for T and N staging by SL. Preoperative T and N staging has a solid limitation due to the discrepancy between clinical and pathological diagnosis,^47^ and SL could not solve this problem. Not only that, SL has a potential disadvantage for T and N staging different from imaging, because it cannot provide complete exploration of the primary lesion and regional lymph nodes. They also reported the accuracy for M staging with overall accuracy, sensitivity and specificity as 85%‐98.9%, 64.3%‐94% and 80%‐100%, respectively. In this analysis, M (distant metastases) included peritoneal dissemination and liver metastases. Small‐sized liver metastases, especially located on the liver surface, are sometimes detected during SL. Recently enhanced magnetic resonance imaging (MRI)^48^ has been able to detect liver metastases with high accuracy, so now the main purpose of SL is the detection of peritoneal disease. The sensitivity of peritoneal disease detection reflects “false negativity”, which is a focus of this review. The specificity may be almost 100%, with the exception of a small number of cases with some difficulty of pathological confirmation.

Patient selection for SL is still controversial. According to the results of the REGATTA trial,^49^ palliative gastrectomy for patients with peritoneal dissemination is not justified, so the detection of peritoneal dissemination during SL is very important for avoiding non‐therapeutic laparotomy and shortening the period until the start of chemotherapy with its lower invasiveness. Yamagata et al^19^ reported an interval of 19.5 days. Almost all advanced gastric cancer patients may be candidates for SL, but it is not a realistic goal. Li et al from the USA reported the problem of cost‐effectiveness.^50^ Accordingly, advanced gastric cancer patients with a high possibility of peritoneal dissemination among clinically P0 patients should undergo SL. Table 1 lists the reported indications for SL studied by multivariate analysis or validation. From these reports, “poorly differentiated adenocarcinoma,” “linitis feature, type 4”, “large sized type 3,” and “equivocal CT findings for peritoneal dissemination” are candidate clinical factors for the indication of SL. A combination of factors may be a good indication, but the accuracy of the indication has, so far, not been discussed in review articles. If the indication for SL is widely defined for advanced tumors, SL can detect many cases of occult peritoneal disease and results in high sensitivity. If the indication is limited, it provides low sensitivity and high specificity. It is a “trade‐off”. If widely defined, a high proportion of all advanced cases are candidates for SL. This introduces the problems of “many negative SL” and “cost‐effectiveness”. Adversely, the population that does not need SL must be carefully inspected.

**Figure 2:**
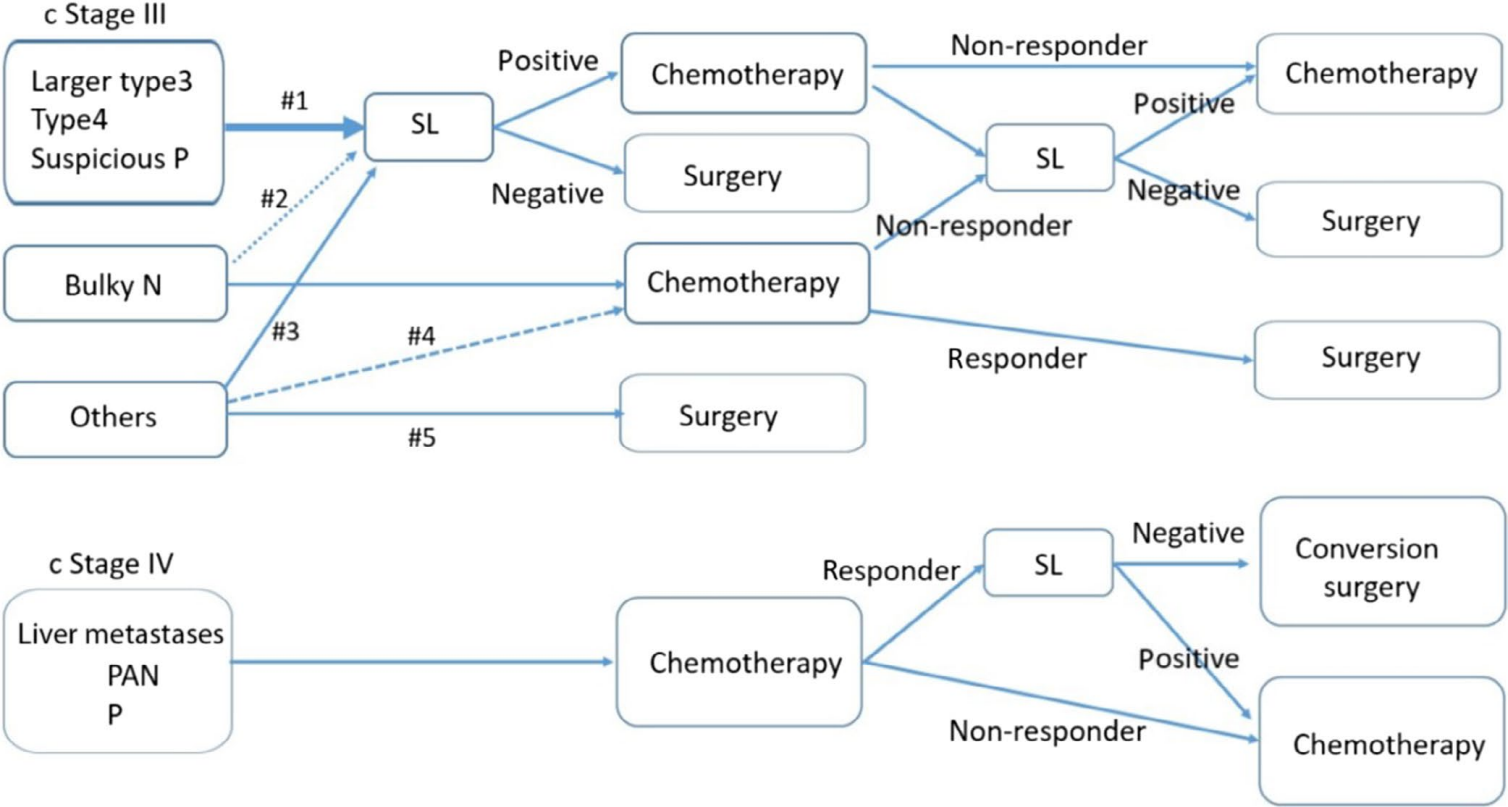
Therapeutic algorithm including staging laparoscopy. Positive, p1 and/or cy1; negative, p0 and cy0. #1, strongly recommended; #2, it can be avoided; #3, in some cases, staging laparoscopy (SL) is recommended; #4, neoadjuvant chemotherapy (NAC) is still controversial; #5, immediate surgery. P, peritoneal dissemination; PAN, para aortic lymph node

The therapeutic strategy in Japan (JCOG0405) for patients with bulky N and PAN is NAC followed by extended surgery. In this clinical trial, SL was mandatory to avoid occult peritoneal disease. Irino et al^18^ reported that 21% of this patient group was positive for peritoneal disease. Accurate information about various type of metastases at the pretreatment stage is important, but initial SL can be avoided for patients with bulky N and PAN. After chemotherapy, SL is needed for decision making about surgery. When NAC is more widely used in patients with clinical stage III gastric cancer,^51^ SL after chemotherapy is also important for detecting occult peritoneal disease especially in case that NAC is not effective (Figure 2), even if initial SL may be avoided.

“False‐negative SL” is a problem. In many studies from Japanese institutions, the proportion of “false negative” rates was reportedly over 10%. The indication for SL in those studies was large type 3 and type 4 that had a high potential of peritoneal disease, so the incidence of “false negative” rates was high. Hato et al^52^ reported a high incidence of “false negative SL” in JCOG0501 targeting the same population. Careful exploration seems to be important in cases with suspicion of peritoneal dissemination.

In many reports, patients with P0CY1 have a poor prognosis, along with patients with macroscopic peritoneal dissemination.^53^ As Badgwell et al^45^ and Jamel et al^44^ reported, better survival could be expected if NAC successfully changed the status from CY1 to CY0. In this therapeutic strategy, selecting patients with POCY1 at first SL and confirming a good response to NAC at repeat SL is very important. In this sense, SL can provide oncological benefit. However, the prognosis for all patients with POCY1 who undergo NAC (including responders and non‐responders) may still be poor. In JCOG0501, where eligibility criteria were large type 3 and type 4, including P0CY1 and localized P1, the survival efficacy of NAC was not justified compared with immediate surgery followed by postoperative chemotherapy.^54^ In a large‐scale retrospective cohort, there was no significant survival difference between NAC and postoperative chemotherapy for patients with P0CY1.^55^ From those reports, the rationale that patients with P0CY1 should undergo NAC may not be justified at this time. If the efficacy of intraperitoneal chemotherapy for peritoneal disease,^56^ including both macroscopic carcinomatosis and positive cytology is established, the importance of SL will be further advanced.

## 5 CONCLUSION

A current literature review suggests that staging laparoscopy is very important for determining the correct therapeutic strategy for the treatment of advanced gastric cancer. (i) Indication for SL is patients with some of the following: “whole stomach,” “type 4 (linitis feature),” “large tumor,” “equivocal CT findings,” and “lymph node swelling”. (ii) The exploration of the entire peritoneal cavity is preferable especially for CY1 patient. (iii) SL has the benefit of detecting occult peritoneal disease, but the false‐negative finding during SL was 0%‐17%. (iv) Yield of SL was higher in gastric cancer compared with esophagogastric junctional tumor. (v) SL‐related complications were estimated to occur in 0.4%. (vi) Repeat SL is important after chemotherapy, especially to enable decision‐making on the need for conversion surgery. (vii) Selection of the “good responder” after chemotherapy for CY1 patients can provide oncological benefit.

## DISCLOSURE

Conflicts of Interest: Author declares no conflicts of interest for this article.
